# Incidence and outcomes of influenza-associated pulmonary aspergillosis and the role of antifungal prophylaxis: a structured literature review

**DOI:** 10.1186/s13054-026-05975-1

**Published:** 2026-03-26

**Authors:** Sarah Sedik, Daniel Felber, Peter Schellongowski, Helmut J.F. Salzer, Romuald Bellmann, Tina Muhr, Julia Auer, Peter Krippl, Martin Lux, Paul Zajic, Markus Werner, Norbert Bauer, Norbert Watzinger, Günther Mesaric, Yasmin Tinawi, Karl Dichtl, Stella Wolfgruber, Subhra Biswas, Jürgen Prattes, Simon Feys, Frank L. van de Veerdonk, Jannes Heylen, Joost Wauters, Agostinho Carvalho, Marius Huguet, Vincent Augusto, Martin Hoenigl

**Affiliations:** 1 Department of Internal Medicine, Division of Infectious Diseases, Medical University of Graz, Excellence Centre for Medical Mycology (ECMM), Graz, Austria; 2 Department of Medicine I, Medical University Vienna, Intensive Care Unit 13i2, Vienna, Austria; 3 Division of Infectious Diseases and Tropical Medicine, Department of Internal Medicine 4 Pneumology, Kepler University Hospital, Linz, Austria. Medical Faculty, Johannes Kepler University, Linz, Austria, Ignaz-Semmelweis-Institute, Interuniversity Institute for Infection Research, Vienna , Austria; 4 Division of Intensive Care and Emergency Medicine, Department of Internal Medicine I, Medical University of Innsbruck, Innsbruck, Austria; 5 LKH Graz II, location West, Internal Medicine 2, Graz, Austria; 6 Internal Medicine, LKH Eastern Styria, Fürstenfeld, Austria; 7 Department of Anesthesiology and Intensive Care Medicine, Medical University of Graz, Graz, Austria; 8 Internal Medicine, LKH Eastern Styria, Hartberg, Austria; 9 Internal Medicine, LKH Eastern Styria, Feldbach, Austria; 10 Diagnostic & Research Institute for Hygiene, Microbiology, and Environmental Medicine, Medical University of Graz, Graz, Austria; 11 The Ignaz Semmelweis Institute, Division of Infectious Diseases, Host-Fungal Pathogen Interaction Group, Translational Medical Mycology Research Unit, European Confederation of Medical Mycology Excellence Center for Medical Mycology, Medical University of Graz, Graz, Austria; 12 Medical Intensive Care Unit, Department of General Internal Medicine, University Hospitals Leuven, Leuven, Belgium; 13 Department of Microbiology, Immunology and Transplantation, KU Leuven, Leuven, Belgium; 14 Department of Internal Medicine and Radboudumc Community for Infectious Diseases (RCI), Radboud University Medical Center, Nijmegen, the Netherlands; 15 Life and Health Sciences Research Institute (ICVS), School of Medicine, University of Minho, Braga, Portugal; 16 ICVS/3B’s - PT Government Associate Laboratory, Guimarães/Braga, Portugal; 17 Center for Biomedical Engineering (I4S Department), Mines Saint-Etienne, Saint-Etienne, 42023 France; 18 Mines Saint-Etienne, Univ Clermont Auvergne, INP Clermont Auvergne, CNRS, UMR 6158 LIMOS, Saint-Etienne, France; 19 BioTechMed Graz, Graz, Austria

**Keywords:** IAPA, Influenza, Aspergillosis, Aspergillus, Prophylaxis

## Abstract

**Background:**

Influenza-associated pulmonary aspergillosis (IAPA) is a frequent and often fatal complication of severe influenza in critically ill patients. Despite increased recognition, its real global burden and optimal prevention strategies remain uncertain.

**Methods:**

A structured literature review of studies published between January 2000 and June 2025 was conducted to summarize IAPA incidence, fatality rate and study heterogeneity. Continuous variables were summarized as trimmed medians (central 80%) with bootstrapped 95% confidence intervals. Between-study heterogeneity and methodological diversity were quantified using the I² and Shannon diversity indices. Non-parametric tests assessed subgroup differences and correlations.

**Results:**

Fifty-three studies (527,475 patients) were included. The trimmed median IAPA incidence was 14.55% (95% CI, 11.16–19.21), and the case-fatality rate was 50.00% (95% CI, 42.86–58.54). IAPA occurred more frequently in ICU-only cohorts (*p* = 0.011) and was associated with higher mortality (*p* = 0.025). Heterogeneity across studies was substantial (I² = 96.1% for incidence; 79.6% for fatality), reflecting differences in diagnostic definitions and patient selection. Universal antifungal prophylaxis, including posaconazole, did not confer a significant survival benefit.

**Conclusions:**

IAPA affects approximately one in six critically ill influenza patients and carries a mortality of around 50%. Rather than a one-size-fits-all approach, future studies are needed to evaluate more individualized therapy concepts that account for patient- and disease-specific factors and may improve overall outcomes.

**Supplementary Information:**

The online version contains supplementary material available at 10.1186/s13054-026-05975-1.

## Key clinical messages

- IAPA is a frequent and high-mortality complication of severe influenza
- Substantial heterogeneity exists across diagnostic frameworks and study populations
- Current evidence does not support routine universal antifungal prophylaxis
- Early disease onset and host heterogeneity limit prophylactic effectiveness
- Risk-adapted and precision medicine approaches remain investigational
- Genetic susceptibility markers may help identify high-risk patients but require validation

**Supplementary Information:**

The online version contains supplementary material available at 10.1186/s13054-026-05975-1.

## Introduction

Influenza-associated pulmonary aspergillosis (IAPA) has emerged as a serious and often fatal complication among critically ill patients with severe influenza [1–4]. Intensive care unit (ICU) case-fatality rates for IAPA range from 40% to over 50% [5, 6], highlighting the urgent need for improved strategies for early detection and management.

Recent studies report that IAPA develops in approximately 10% to 20% of influenza patients admitted to ICUs [5, 7]. Diagnostic confirmation remains difficult because the infection typically arises early (often within 48 h of ICU admission), and procedures such as bronchoscopies for bronchoalveolar lavage (BAL) and fluid sampling are frequently impractical in patients with unstable respiratory status [2].

A randomized proof-of-concept trial (POSA-FLU) evaluated universal antifungal prophylaxis with posaconazole in critically ill influenza patients [6]. While the study showed a numerical reduction in IAPA incidence (5.4% vs. 11.1%), the difference did not reach statistical significance, likely due to the limited sample size and the high rate of early-onset IAPA in both groups [8]. More recently, a multicenter propensity score–weighted observational study across nine ICUs found that empirical mold-active antifungal treatment (predominantly posaconazole), when initiated within 24 h of ICU admission, significantly reduced IAPA incidence (7.7% vs. 20.4%) but did not improve short- or long-term ICU survival [9]. The study confirmed that most IAPA cases occur within the first 48 h of ICU admission, limiting the effectiveness of delayed therapeutic or prophylactic strategies [9]. These findings highlight the need for more targeted approaches—for example, focusing on well-defined high-risk subgroups and suggest that a more personalized prevention strategy combining early prophylaxis with immunomodulation or immunotherapy [10–12] may hold promise for future prevention and early treatment. Such individualized interventions could improve outcomes while minimizing unnecessary drug exposure and potential resistance development.

While approaches that integrate host factors, immune profiles, and particular genetic predispositions [13] may be most promising to fit the unmet need of identifying high-risk patients who may benefit most from targeted antifungal and immunomodulatory prophylaxis/early treatment, accurate estimates of the epidemiological burden and outcomes of IAPA across centers are needed for designing such studies. To address this need, a structured literature review was conducted to summarize and critically appraise existing evidence [14, 15]. The objective of this structured review was to synthesize current epidemiological data on the incidence and outcomes of IAPA among hospitalized and critically ill influenza patients, with quantitative and qualitative data extraction focusing on (i) the population at risk, (ii) the proportion diagnosed with IAPA, and (iii) the mortality among IAPA cases, alongside diagnostic approaches, study designs, and settings, and (iv) the usage of prophylactic approaches in the therapeutic scheme [16–18].

## Methods

A comprehensive search was performed in PubMed/MEDLINE covering the period from January 1 st 2000 to June 30th 2025. PubMed was chosen for its comprehensive coverage of biomedical and infectious disease literature relevant to the study question [19, 20]. Search terms combined synonyms for influenza and aspergillosis, as well as specific terms for IAPA. The full strategy is provided in the Supplementary Appendix 1 (search strategy). The last search was conducted on October 19, 2025. Two recently published studies after October 19th 2025 were additionally included manually due to their high relevance to the research question [9, 21].

One reviewer independently screened all search results for relevance, with consultation from several co-authors when necessary. Titles and abstracts were assessed for relevance to influenza, aspergillosis, or both. For studies meeting the inclusion criteria, the full texts were reviewed for any exclusion criteria. If multiple exclusion criteria were applicable, the first identified criterion was recorded as the primary cause for exclusion.

### Eligibility criteria

Inclusion criteria comprised studies published in English or German that focusprimarily on influenza, aspergillosis, or the combination of both. During the initial screening step, records in which the acronym “IAPA” referred to concepts unrelated to influenza-associated pulmonary aspergillosis were classified as ambiguous acronym matches and were excluded in subsequent review stages.

Exclusion criteria included case reports or case series with fewer than five patients, as well as editorials, commentaries, surveys, guidelines, and other position papers lacking epidemiological data. Studies were also excluded if they did not address the combination of influenza and aspergillosis, if they focused on aspergillosis in a broader context, if IAPA was only described as a subgroup, if exact IAPA case numbers could not be determined, or if the work involved exclusively animal, in vitro, or material studies. In addition, studies without the intention of demonstrating a true infection (i.e. focusing exclusively on mycological test results and not taking into account clinical and radiological factors), and those referring exclusively to already included studies were excluded.

### Data extraction

Data were extracted using a standardized, predefined form and included bibliographic information (author, title, journal, country, and year of publication), the number of patients at risk (influenza cases), the number of *Aspergillus* infections (classified as invasive rather than colonizing), IAPA fatality rate or mortality and the diagnostic criteria used for defining influenza and *Aspergillus* infection.

Additional variables collected were the study setting (ICU-only or hospital-wide), hospital type, and key study design characteristics, including study type, prospective vs. retrospective design, single- versus multicenter setting, and the time frame of patient inclusion.

For the purposes of this review, the sample size was defined as the total number of influenza patients among whom Aspergillus infections were reported. IAPA was defined as cases classified as *Aspergillus* infection by the original study authors. For mortality or IAPA fatality rate, the longest available follow-up period reported by each study was used. Diagnostic criteria were assigned according to those applied to the majority of IAPA cases; where multiple criteria were used without further specification, categories were recorded as combined.

If information in the predefined data category was missing, it was recorded as not mentioned. An exception was made for the category “proven/probable/both aspergillosis cases”: when the study authors had applied an established diagnostic algorithm but did not specify the breakdown, we assumed that both proven and probable cases were included [9, 22].

ICD coding–based studies were defined as studies identifying influenza or aspergillosis cases solely through International Classification of Diseases (ICD) diagnostic codes (e.g., ICD-9 or ICD-10), without individual clinical or microbiological case verification [23].

For consistency, the term incidence was used throughout the manuscript to describe included IAPA cases under the assumption of a newly acquired disease during the influenza infection period, whereas prevalence was not used as the included studies primarily reported newly diagnosed cases during the observation period rather than chronic diseases.

### Statistics

All statistical analyses were conducted using IBM SPSS Statistics Version 29 (IBM Corp., Armonk, NY, USA). Descriptive statistics were used to summarize study characteristics and outcome measures. Due to the high heterogeneity within included studies, robust estimators against outliers were used to summarize central tendencies. Continuous variables were reported as trimmed medians (central 80% of observations, based on study sample sizes; 10% trimmed from each tail) with interquartile ranges (IQR), supplemented by non-parametric bootstrapped 95% confidence intervals (1,000 resamples; percentile method). Categorical variables were summarized as frequencies and percentages. A formal meta-analysis was considered but not conducted because of pronounced heterogeneity and inconsistent diagnostic definitions among studies. Diagrams and graphics were generated in IBM SPSS Statistics Version 29 (SPSS Inc., Chicago, IL, USA) and MapChart (n.d.). Trimmed medians were not applied to correlation analyses or graphical representations. Results are reported with a precision appropriate to the respective measure.

Between-study heterogeneity was quantified using the I² statistic, calculated for the proportional outcomes IAPA incidence and IAPA fatality rate. I² represents the proportion of total variation across studies attributable to true heterogeneity rather than random error, with 25%, 50%, and 75% conventionally interpreted as low, moderate, and high heterogeneity, respectively. I² was not applied to count-based variables (e.g., sample size, number of IAPA cases, number of deaths), as these variables serve as denominators or weight components rather than independent outcomes.

To assess the structural and methodological diversity among studies, the Shannon diversity index was calculated as

$$\:H\:=\:-\varSigma\:\:p_i\:ln\:p_i$$

where $p_i$ denotes the proportion of studies within category *i*. Shannon indices were computed separately for continent, aspergillosis diagnostic criteria, study setting (ICU-only vs. hospital-wide), and center structure (single- vs. multicenter). Higher *H* values indicate greater diversity, with the theoretical maximum given by ln(*k*), where *k* is the number of categories.

While I² quantifies statistical heterogeneity in effect estimates, the Shannon index captures methodological and structural diversity across studies, providing a complementary measure of between-study variability. This dual approach allowed assessment of both statistical heterogeneity and methodological diversity, which together reflect the multifactorial variability characteristic of IAPA research.

Given the non-normal distribution of study-level data and unequal group sizes, non-parametric methods were used for all between-group comparisons. The Mann–Whitney U test was applied for two-group comparisons (e.g., ICU vs. hospital-wide), and the Kruskal–Wallis H test for comparisons across multiple categories (e.g., continents, diagnostic criteria). Associations between continuous study variables were analyzed using Spearman’s rank correlation coefficient (ρ). As an internal validity check, correlations between fundamental count variables (sample size, IAPA cases, and deaths) were examined to confirm internal data consistency and expected positive associations. All statistical tests were two-sided, and *p* < 0.05 was considered statistically significant. Given the exploratory nature of the analysis, no correction for multiple comparisons was applied.

To confirm that the observed association between setting and IAPA fatality rate was not influenced by extreme values, an outlier analysis was performed. Studies limited to post-mortem examinations, and therefore restricted to fatal cases by design, were excluded from quantitative analyses on fatality rates. The relationship between sample size and IAPA incidence was visualized in a scatterplot using locally estimated scatterplot smoothing (LOESS), a non-parametric method that fits local regressions to subsets of the data to capture non-linear patterns. LOESS smoothing was applied for descriptive visualization only and was not used for statistical inference.

To assess potential small-study effects and publication bias, we created an inverse standard error (SE) funnel plot on logit-transformed incidence with the exclusion of ICD coded-based IAPA-studies. Study precision, as inverse SE, was used as a proxy for study size. Logit transformation for incidence estimates was calculated to reduce skewness and stabilize variance, thereby improving compatibility with methods assuming approximate normality of effect estimates, as required for funnel plot analyses. In addition, Egger’s regression test were applied to further evaluate funnel plot asymmetry for the full dataset and with the exclusion of ICD-coded studies.

As an additional subgroup of interest, we examined studies evaluating the impact of antifungal prophylaxis in patients with influenza. Because only a limited number of studies addressed this topic, we synthesized the findings narratively rather than performing a quantitative analysis.

### Reporting standards

Although this review differs from a classic systematic review by relying on a single bibliographic database and one primary reviewer, it followed the PRISMA guidelines for systematic reviews as a reporting framework to ensure adherence to established quality standards [24]. The guidelines were adapted where justified deviations were necessary. Detailed descriptions and rationales for these deviations are provided in the Supplementary Appendix 2 (PRISMA checklist).

A PRISMA 2020-style flow diagram was used to visualize the study selection process, including inclusion and exclusion decisions at each screening stage.

## Results

### Study selection and characteristics

**Fig. 1:**
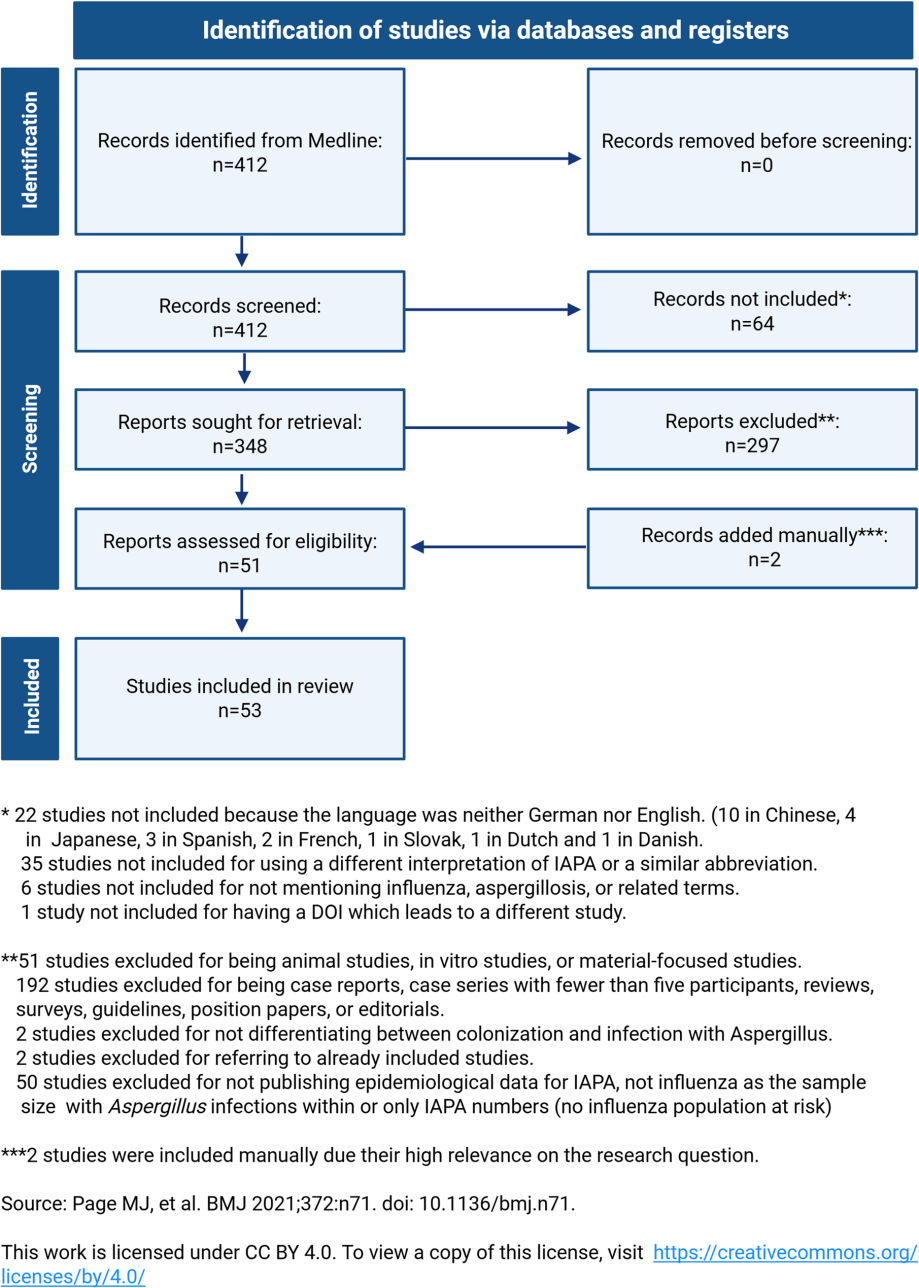
PRISMA diagram of study selection

The study selection process is summarized in Fig. 1. A total of 412 records were identified, of which 348 were retrieved for full-text screening. Studies that appeared to meet the inclusion criteria but for which full-text access was unavailable were excluded from the statistical analysis. After applying all predefined inclusion and exclusion criteria, 51 studies were finally included. Two studies were added manually, making a total of 53 studies in the final analysis. Across all included studies, the total study population comprised 527,475 patients. If ICD coding-based studies were excluded, 12,066 patients would be analyzable in this review.

**Fig. 2:**
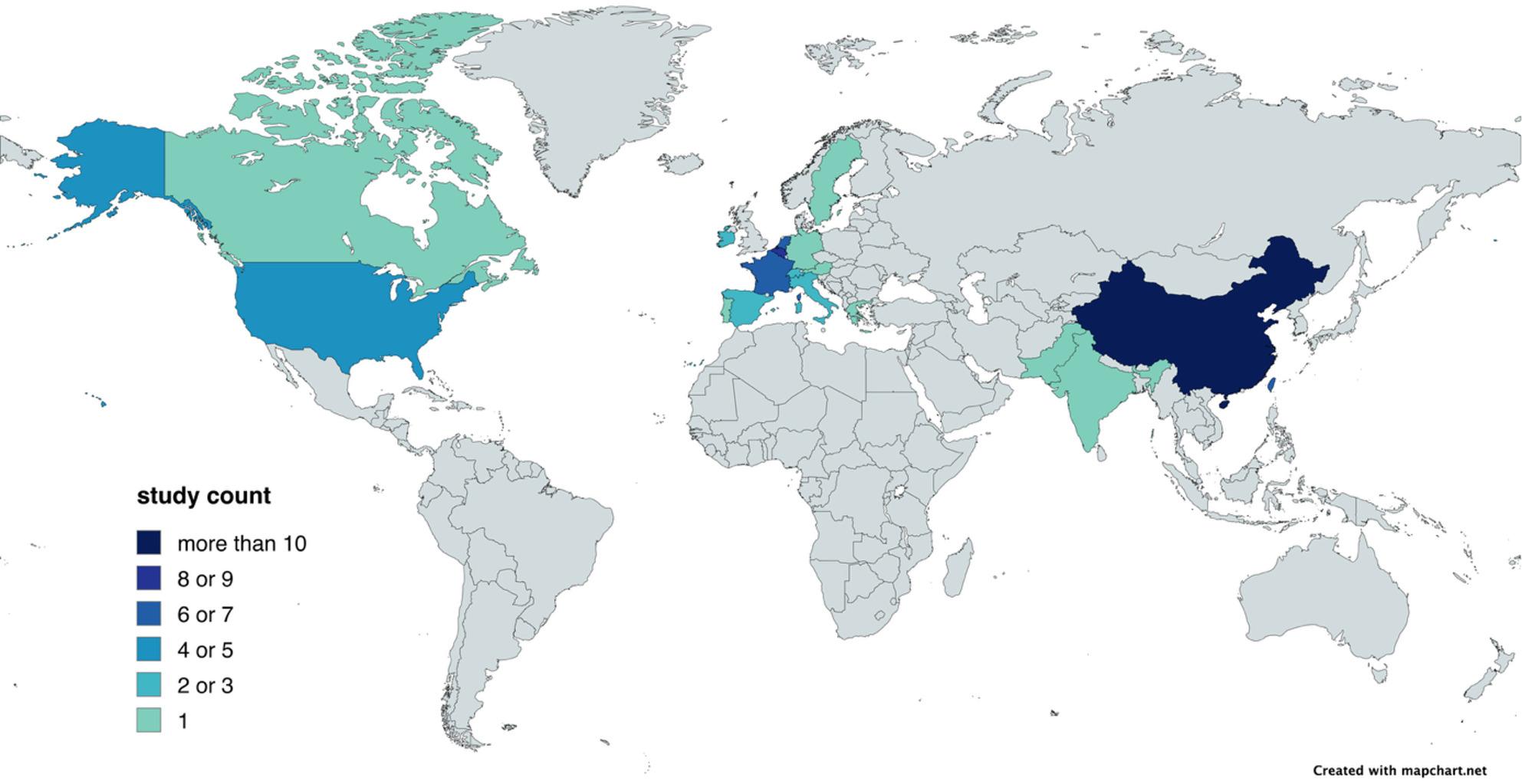
Geographic distribution of included studies. Countries contributing to the included studies are shown in color, with the color intensity indicating the number of studies per country (see scale). A total of 18 countries contributed data, with most studies originating from Europe, followed by Asia and North America. The highest number of studies were conducted in China (*n* = 11), Belgium (*n* = 9) and Taiwan (*n* = 7)

All included studies are shown in Supplementary Appendix 3 [1, 3, 6, 8, 9, 21, 25–71]. The studies were published between 2012 and 2025 and covered data from three continents: Europe (*n* = 28), Asia (*n* = 20), and North America (*n* = 5). An overview of the contributing countries is visualized in Fig. 2; Table 1. Most studies were conducted in ICU settings (*n* = 37; 70%), while the remaining studies focused on hospital-wide cohorts (*n* = 16, 30%). Information on study design, center structure, and observation type was extracted across studies and is summarized in Supplementary Appendix 3.

**Table 1:** Studies per country (multiple entries per study possible)

Country	Count
**China**	11
**Belgium**	9
**Taiwan**	7
**France**	6
**The Netherlands**	6
**United States**	4
**Switzerland**	3
**Ireland**	2
**Italy**	2
**Spain**	2
**Austria**	1
**Canada**	1
**Germany**	1
**Greece**	1
**India**	1
**Pakistan**	1
**Portugal**	1
**Sweden**	1

**Fig. 3:**
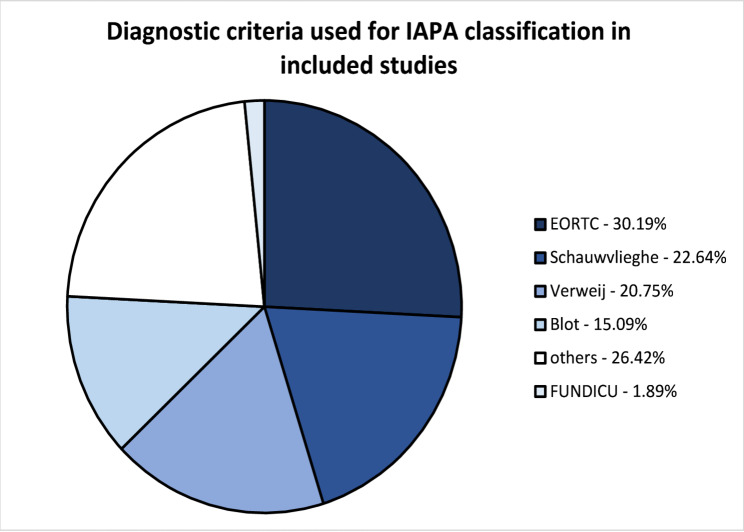
Overview of used diagnostic criteria and methods across included studies. When multiple criteria were used in the same study, each criterion was counted separately. Shown numerical percentages are based on the total number of studies (*n* = 53) and do not represent proportions relative to each other

Diagnostic definitions for invasive aspergillosis varied substantially across studies. The most frequently applied definitions were those of the EORTC/MSG (European Organization for Research and Treatment of Cancer/Mycoses Study Group) [22, 72] and Schauwvlieghe et al. [5], followed by the criteria proposed by Verweij et al. [2] and Blot et al. [73], and the most recent FUNDICU criteria [74], as well as mixed or study-specific diagnostic approaches. Influenza infection was predominantly confirmed by polymerase chain reaction (PCR) testing, either alone or in combination with other diagnostic methods. A graphical overview of the distribution of diagnostic criteria and methods is provided in Fig. 3. Shown percentages are based on the proportion of included studies in which each criterion was used, when multiple criteria were used, each one was counted separately. A more detailed overview is provided in Supplementary Appendix 3.

Across the central 80% of studies (trimmed analysis, based on sample size), the median study sample size was 124 patients (IQR = 141; range = 24–693). In total, the combined dataset of the central 80% of studies included 7,285 influenza patients, of whom 945 had documented IAPA. The trimmed median number of IAPA cases per study was 17 (95% CI 9–21; IQR = 21), and the trimmed median number of deaths among IAPA patients was 8 (95% CI 4–13; IQR = 11).

Trimmed IAPA incidence values ranged from 1.91% to 46.15% (median = 14.55%; 95% CI 11.16–19.21), and fatality rates among IAPA patients ranged from 0.00% to 100.00% (median = 50.00%; 95% CI 42.86–58.54). Confidence intervals were derived using non-parametric bootstrap resampling (1,000 iterations).

### Study heterogeneity and subgroup analyses

Marked heterogeneity was observed for both proportional outcomes. The I² statistic indicated substantial inter-study variability for IAPA incidence (I² = 96.1%) and fatality rate (I² = 79.6%).

To quantify methodological and structural diversity, Shannon diversity indices (H) were calculated for key categorical variables. The resulting indices indicated moderate to high diversity across:

- Continent (H = 0.928),
- IAPA diagnostic criteria (H = 1.800),
- Study setting (ICU-only vs. hospital-wide, H = 0.612), and.
- Center type (single- vs. multicenter, H = 0.693).

Subgroup analyses revealed no significant differences in IAPA incidence across continents (Kruskal–Wallis H = 2.853, *p* = 0.240) and no significant differences in fatality rates (H = 2.398, *p* = 0.301).

When grouped by diagnostic criteria, IAPA incidence again showed no significant variation (H = 4.257, *p* = 0.642). Also, fatality rates did not differ significantly across diagnostic systems (H = 10.083, *p* = 0.073).

By study setting, IAPA incidence was significantly higher in ICU-only studies compared with hospital-wide cohorts (16.67% [IQR 13.39%] vs. 5.93% [IQR 16.11%]; Mann–Whitney U = 165.0, *p* = 0.011). After excluding two studies designed to include only post-mortem cases (reporting 100% mortality per design, one ICU-only, one hospital-wide), IAPA fatality rates were also significantly higher in ICU-only studies (50.30% [IQR 21.05%] vs. 21.38% [IQR 50.30%]; U = 49.0, *p* = 0.025).

### Correlations and inter-variable associations

Spearman’s rank correlation analyses were performed to explore relationships between key continuous variables. There was no significant correlation between IAPA incidence and fatality rate (ρ = 0.208, *p* = 0.224). IAPA incidence showed a significant negative correlation with sample size (ρ = −0.607, *p* < 0.001), indicating that smaller studies tended to report higher incidence estimates. No significant correlation was observed between publication year and either IAPA incidence (ρ = −0.086, *p* = 0.542) or fatality rate (ρ = −0.018, *p* = 0.919). Similarly, when using the median year of the study inclusion period instead of publication year, no significant association was found with IAPA incidence (ρ = −0.113, *p* = 0.431) or fatality rate (ρ = 0.091, *p* = 0.611).

When stratified by study setting, the inverse correlation between study size and IAPA incidence remained significant in both ICU-only cohorts (ρ = −0.558, *p* < 0.001) and hospital-wide studies (ρ= −0.739, *p* < 0.001).

### Multivariable linear regression analysis

To assess the independent associations of diagnostic criteria with IAPA outcomes while adjusting for other factors, two multivariable linear regression models were calculated. The first model used IAPA incidence as the dependent variable, and the second used IAPA fatality rate. Diagnostic criteria (EORTC/MSG, Verweij, Schauwvlieghe, and Blot) [2, 22, 54, 72, 73] were entered as independent categorical variables.

**Fig. 4:**
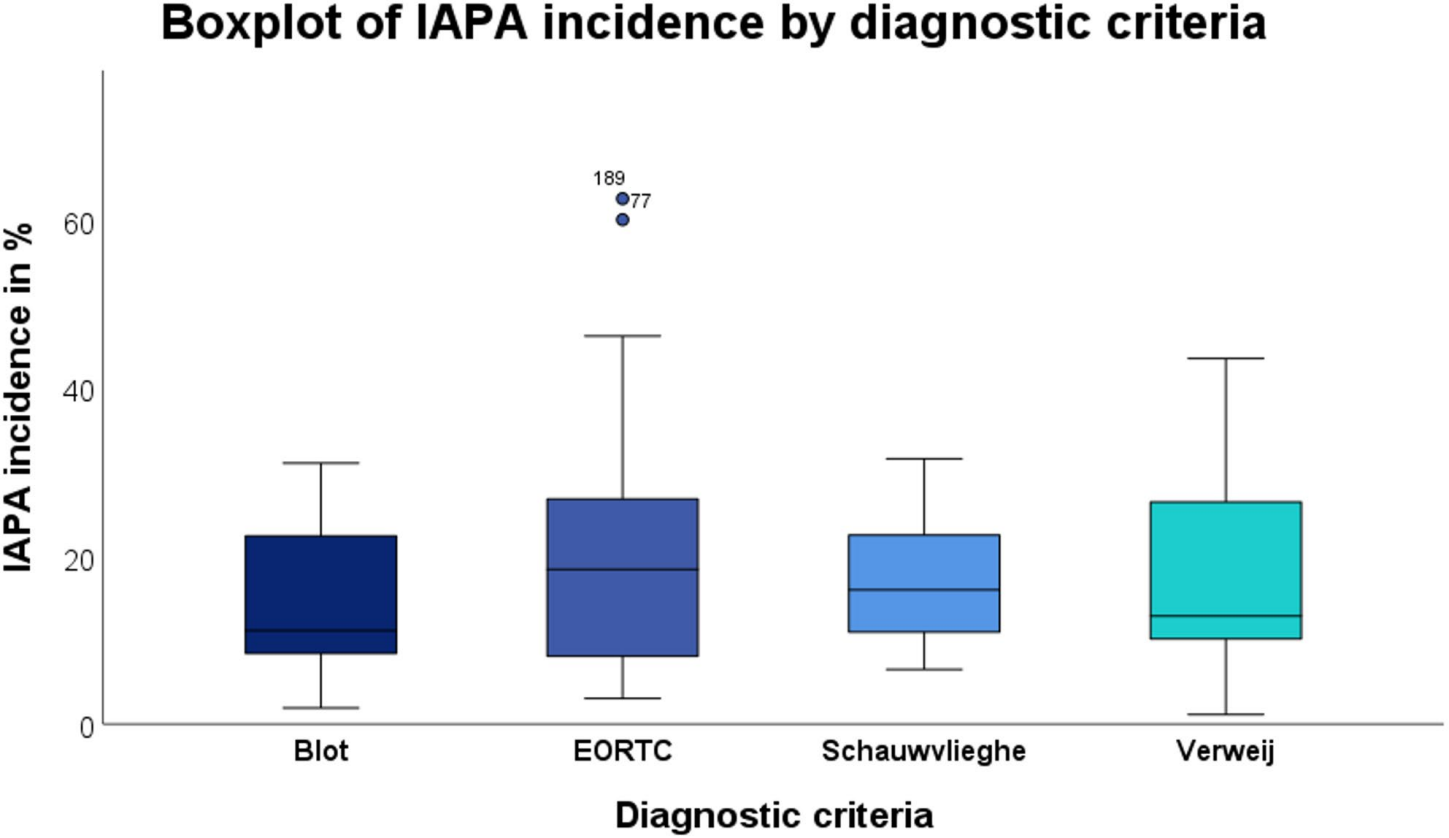
Boxplot overview on IAPA incidence across different diagnostic criteria. Circles indicate mild outliers (1.5–3.5 x IQR). No statistically significant association was observed between diagnostic criteria and IAPA incidence (p-values ranged from 0.064 to 0.536). Multiple entries were possible for studies applying more than one diagnostic criterion

For the model examining IAPA incidence (Fig. 4), none of the diagnostic criteria showed a statistically significant independent association. The regression coefficients (B) ranged from − 4.66 (Blot criteria) to + 9.13 (EORTC criteria), with all p-values > 0.05 (EORTC: B = 9.13, *p* = 0.064; Verweij: B = 6.18, *p* = 0.264; Schauwvlieghe: B = 3.40, *p* = 0.526; Blot: B = −4.66, *p* = 0.412).

**Fig. 5:**
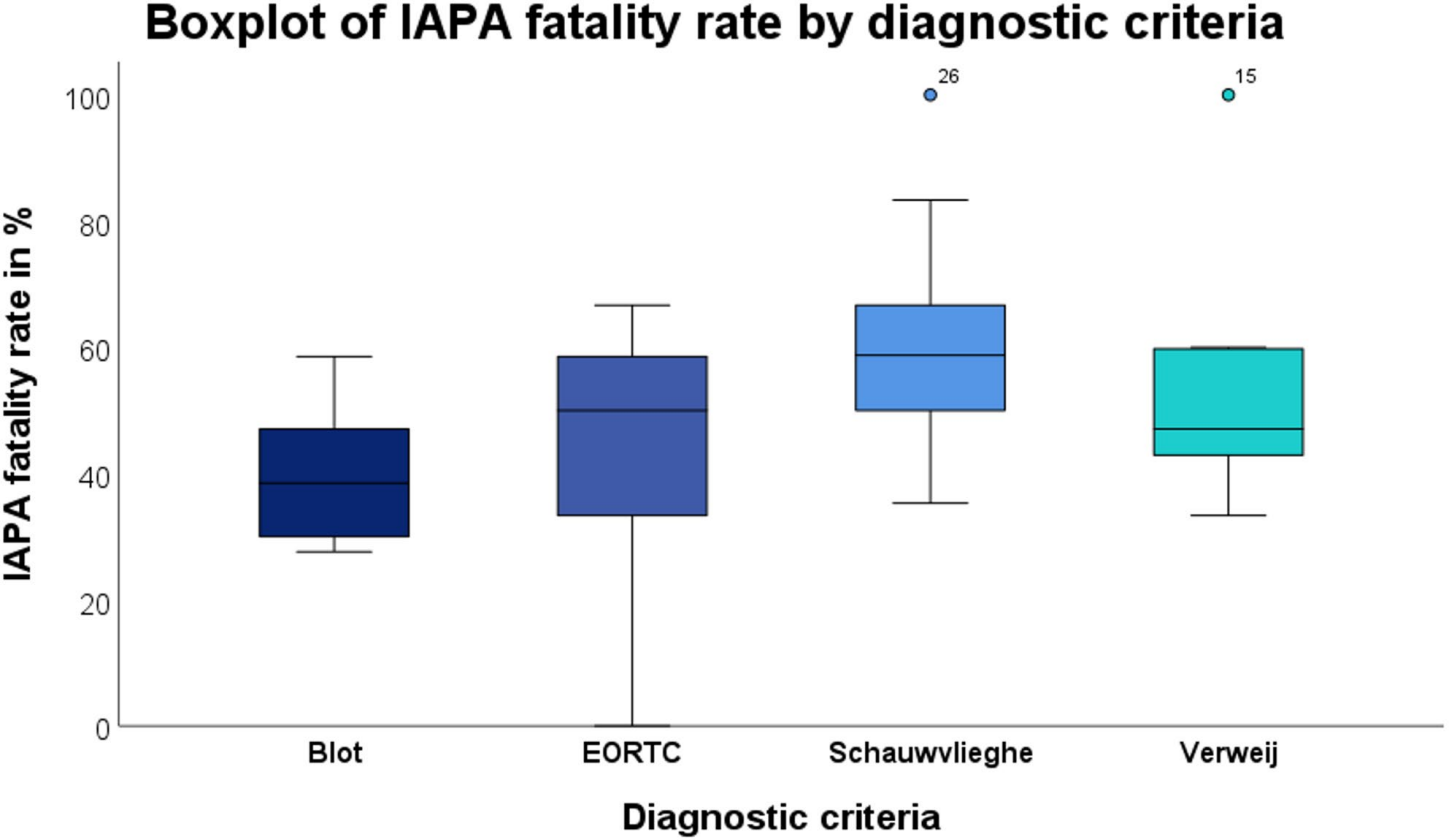
Boxplot overview on IAPA fatality rate across different diagnostic criteria. Circles indicate mild outliers (1.5–3.5 x IQR). No statistically significant association was observed between diagnostic criteria and IAPA fatality rate (p-values ranged from 0.054 to 0.943). Multiple entries were possible for studies applying more than one diagnostic criterion

In the model for IAPA fatality rate (Fig. 5), there was a trend towards higher mortality in studies applying the Schauwvlieghe (B = 21.49, *p* = 0.058) and Verweij criteria (B = 16.89, *p* = 0.168); however, neither association reached statistical significance.

### Graphical representation of findings

**Fig. 6:**
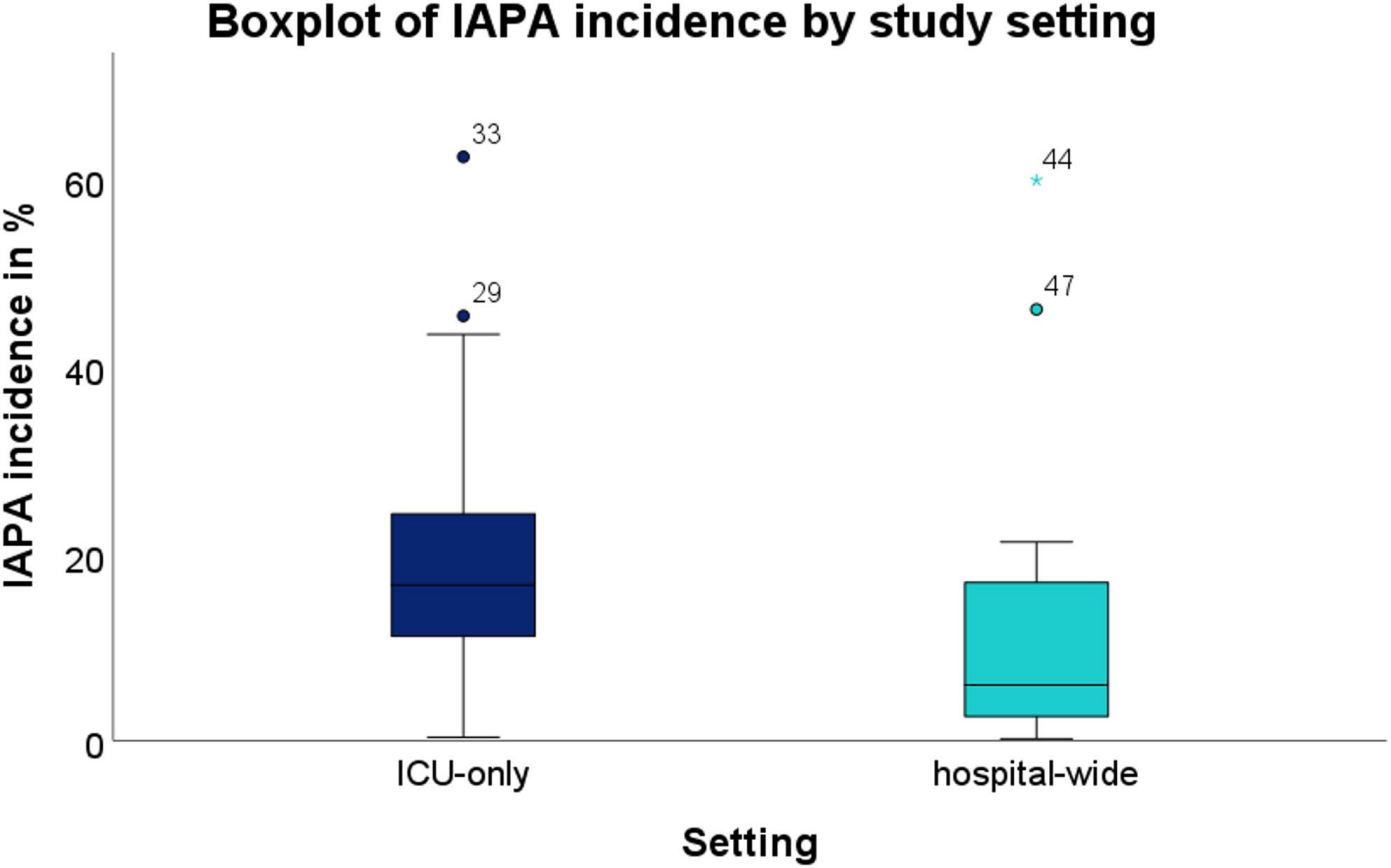
Boxplot of IAPA incidence by study setting. IAPA incidence was significantly higher in ICU-only studies than in hospital-wide studies (*p* = 0.012). Circles indicate mild outliers (1.5 to 3 × IQR), and asterisks indicate extreme outliers (> 3 × IQR), as defined by SPSS

**Fig. 7:**
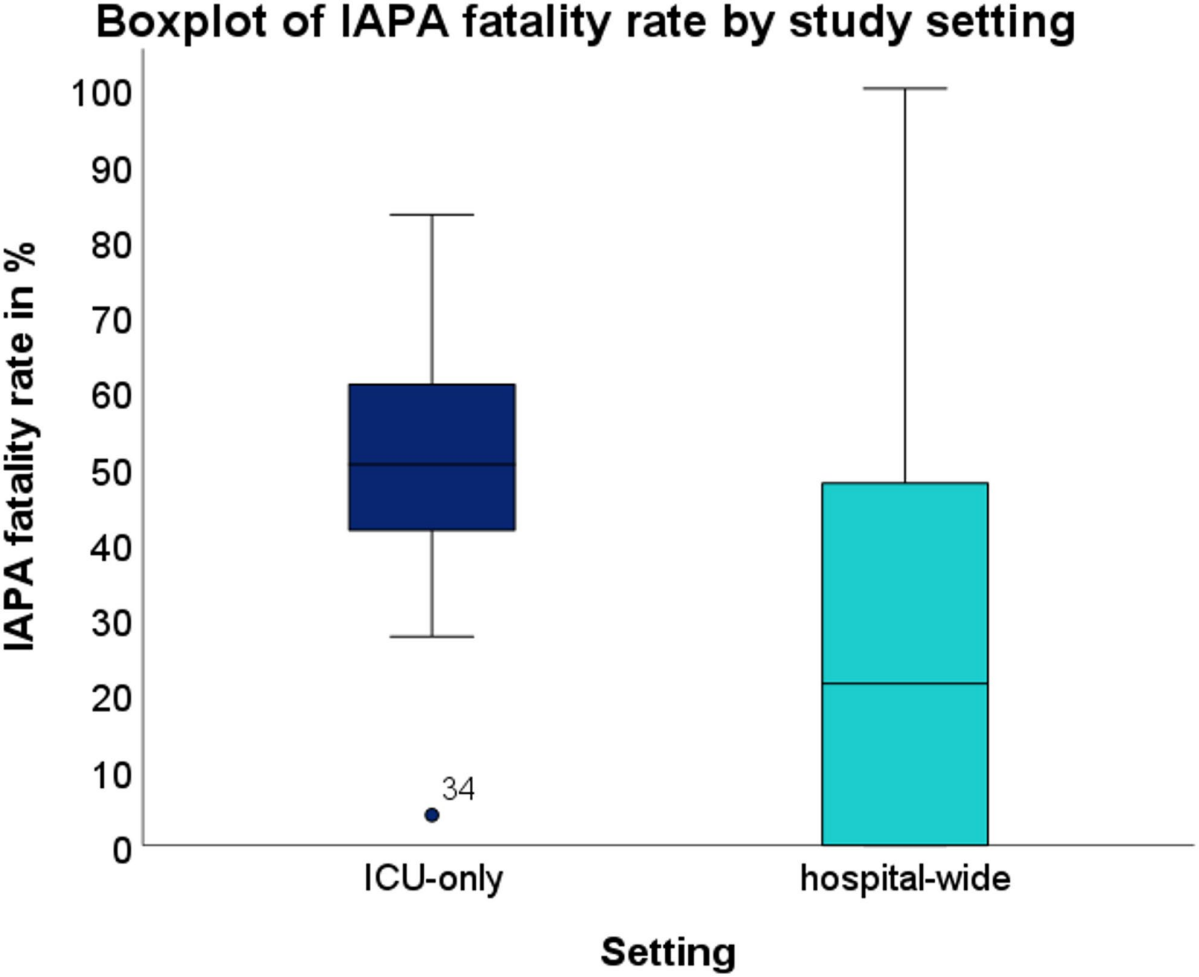
Boxplot of IAPA fatality rate by study setting. The fatality rate among IAPA cases was significantly higher in ICU studies compared to hospital-wide studies (*p* = 0.028). In each group, one study reporting 100% fatality was excluded, as only fatal cases were observed in those studies per design. Circles indicate mild outliers (1.5 to 3 × IQR)

Visual analyses were performed to illustrate the distribution and relationships of key study variables. Boxplots (Figs. 6 and 7) compared IAPA incidence and fatality rates across major subgroups, including study setting (ICU-only vs. hospital-wide), continent, and diagnostic criteria.

**Fig. 8:**
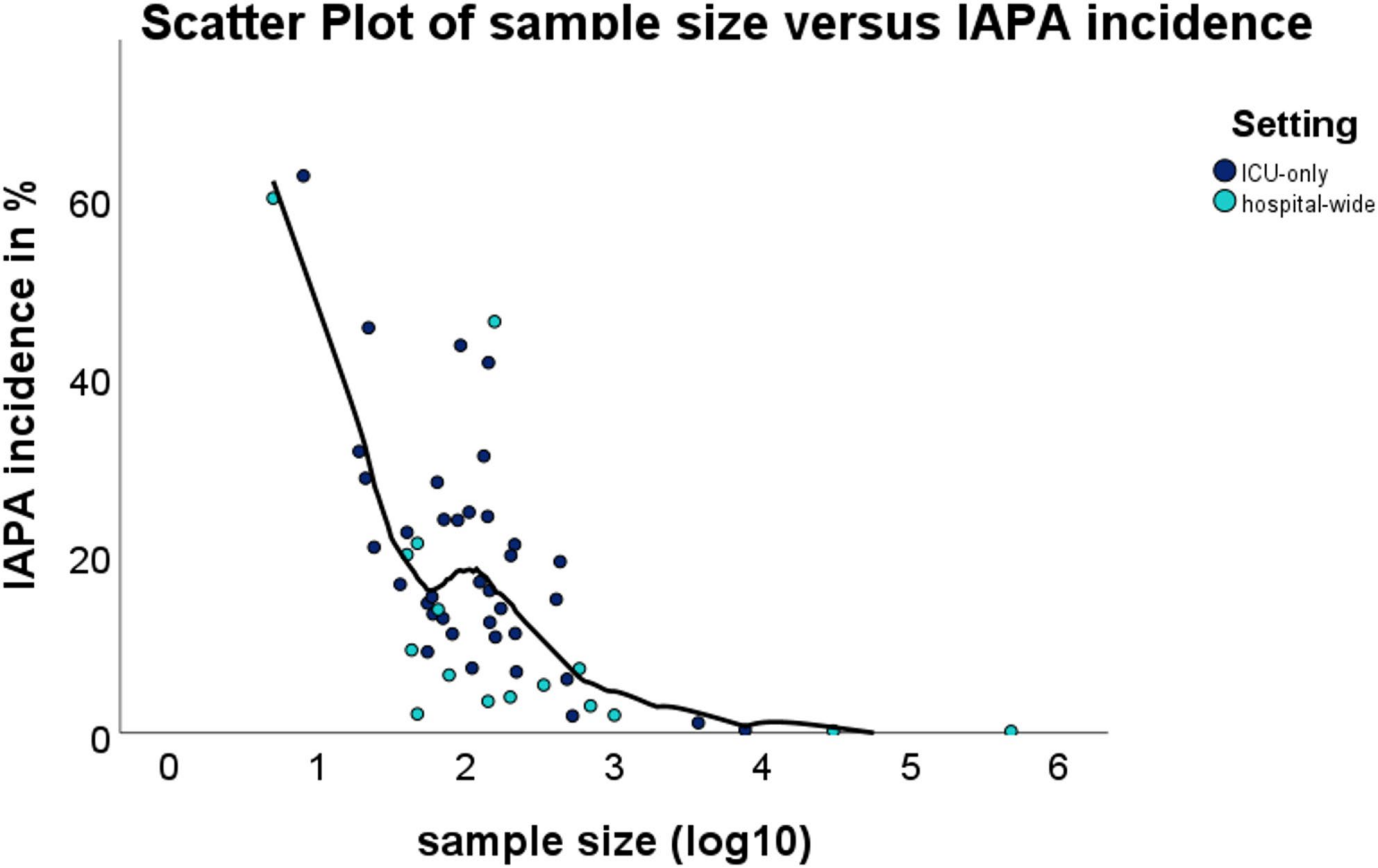
Scatter plot showing inverse correlation between sample sizes versus IAPA incidence; LOESS trend line showing the relationship while accounting for skew. A significant inverse correlation was observed between study sample size and IAPA incidence (ρ= −0.607, *p* < 0.001)

A scatterplot (Fig. 8) depicting study sample size versus IAPA incidence further emphasized the inverse association between cohort size and reported incidence. Smaller studies tended to yield higher incidence estimates, whereas larger cohorts demonstrated more moderate values. The fitted LOESS trend line illustrated this negative relationship while accounting for non-linearity and distributional skew.

**Fig. 9:**
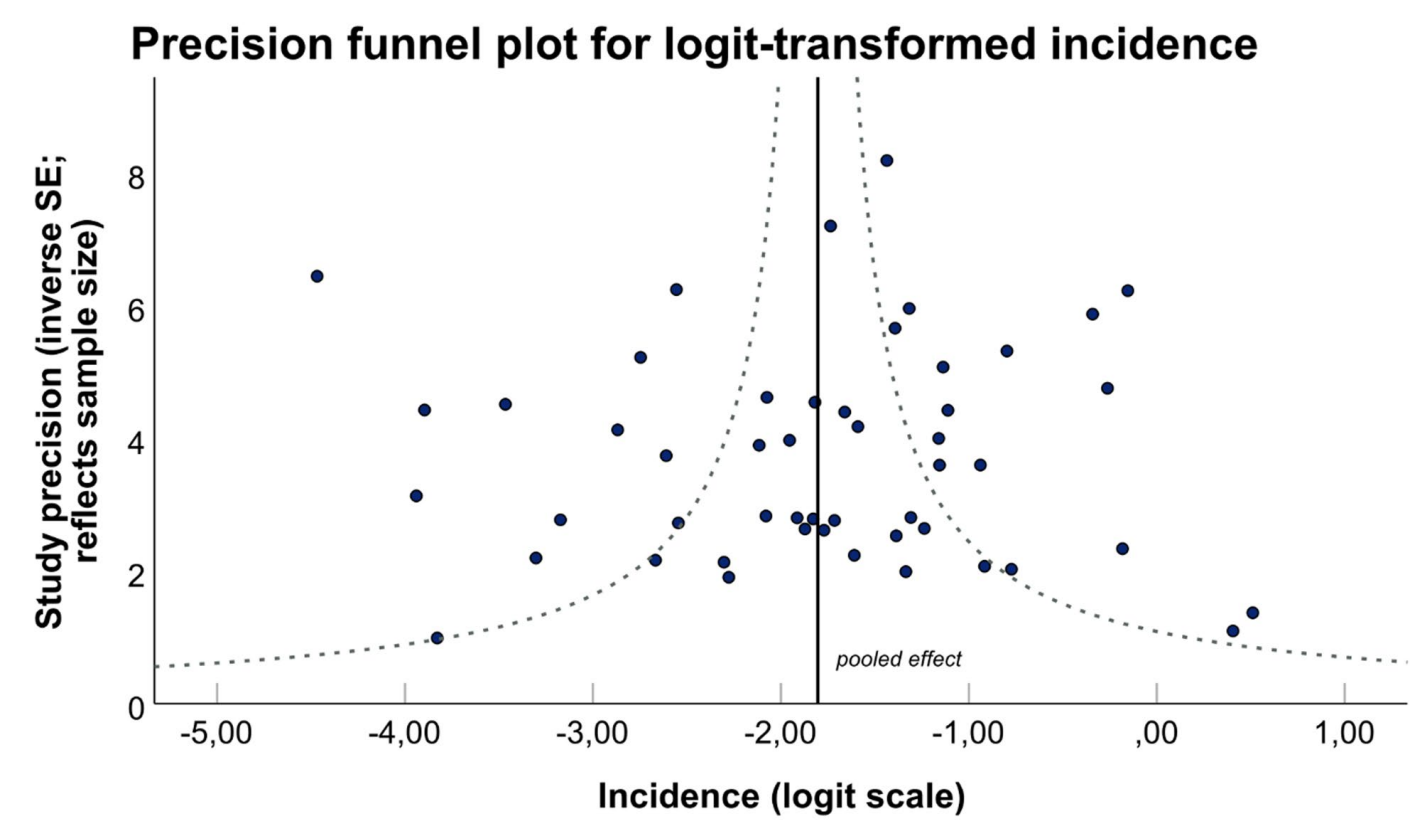
Funnel plot showing study precision (inverse standard error, proxy for study size) against logit-transformed incidence. No significant difference of funnel plot asymmetry was observed (Egger’s regression *p* = 0.878), however, a wide variation in incidence estimates was shown among all studies, independent of study size

A precision funnel plot excluding ICD-coded studies (Fig. 9) was created to investigate potential small-study effects and publication bias, visualizing study precision versus logit-transformed IAPA incidence. Considerable variation in effect estimates was observed even among studies with a high number of influenza cases, without evidence of trends, showing an overall symmetrical study distribution.

To quantify the visual symmetry, Egger’s regression test was performed. While significant asymmetry was observed for all studies (*p* < 0.001), no evidence of asymmetry remained after exclusion of ICD-coded studies (*p* = 0.878).

### Antifungal prophylaxis

Across the three studies investigating antifungal prophylaxis in influenza-associated critical illness, substantial heterogeneity was evident in IAPA incidence, temporal disease dynamics, and clinical outcomes across patient subgroups. In the POSA-FLU randomized trial, IAPA was diagnosed in 24% of 88 enrolled patients, with 71% of cases occurring within 48 h of ICU admission, thereby markedly limiting the window during which prophylaxis could be administered prior to disease onset. There was an approximate 50% relative reduction in IAPA incidence (5.4% vs. 11.1%) in those receiving prophylaxis, though this difference did not reach statistical significance, likely due to sample-size limitations and the high frequency of early-onset IAPA in both arms. 90-day mortality did not differ significantly between the prophylaxis and standard-of-care groups (24.3% vs. 30.6%, *p* = 0.61). Among patients with late-onset IAPA, mortality was 50% (one in two patients) under prophylaxis compared with 75% (three in four patients) under standard care, although interpretation is constrained by the minimal sample size [8]. In a neutropenic cohort during the 2009 influenza pandemic, invasive aspergillosis occurred in three out of five patients, including two breakthrough infections despite adequate azole serum concentrations during prophylaxis. Both patients had an acute myelogenous leukemia as a predisposing underlying disease [62]. In a larger observational cohort, early empirical antifungal therapy was provided in 35% of 172 patients. The 30-day incidence of IAPA was lower in this group compared with patients without early antifungal treatment (7.7% vs. 20.4%). However, mortality at data cutoff remained high (above 60%) and comparable between groups (*p* = 0.522), and neither 30-day nor 90-day ICU mortality differed significantly; mortality was numerically higher among patients receiving prophylaxis [8, 9, 62].

## Discussion

In this review of 53 studies, we observed a substantial IAPA disease burden, particularly in ICU settings. The trimmed median incidence of IAPA was 14.76% (95% CI, 11.14–19.90), and the case-fatality rate was 48.53% (95% CI, 42.86–58.54). Marked heterogeneity was evident across studies, reflecting differences in diagnostic criteria, patient selection, and study setting. Several diagnostic frameworks were applied, including EORTC/MSG [22, 72], Schauwvlieghe et al. [5], Verweij et al. [2], and Blot et al. [73]. While the recent 2024 FUNDICU criteria [74] may overcome this limitation in the future, the previous lack of standardized diagnostic criteria in ICU populations—where histopathological confirmation is rarely feasible—may contribute to misclassification [2, 75]. Subgroup analyses showed that studies conducted exclusively in ICU settings reported significantly higher IAPA incidence and mortality than hospital-wide cohorts [2, 14]. We also observed an inverse association between study size and reported incidence, suggesting, in combination with the conducted funnel plot and Egger’s testing, that more intensive and targeted diagnostic efforts in smaller, dedicated studies may have contributed to higher reported incidence estimates, in which IAPA was more frequently identified through active screening for aspergillosis than in large-scale studies without systematic diagnostic work-up. Collectively, these findings highlight substantial clinical, methodological, and diagnostic heterogeneity across studies, supporting cautious interpretation of pooled epidemiological estimates.

An additional factor contributing to variability across studies is the use of different diagnostic definitions for invasive aspergillosis in ICU patients. Criteria such as the EORTC/MSG definitions, originally developed for immunocompromised populations, may underestimate IAPA in non-neutropenic critically ill patients, whereas ICU-adapted definitions such as the FUNDICU criteria [74], Aspergillus in the ICU (AspICU) and the modified AspICU criteria have been specifically proposed for critical care settings [76]. These differences can influence not only reported incidence but also timing of diagnosis and classification of disease severity, with broader criteria yielding higher case ascertainment and more restrictive ones identifying fewer, more advanced cases [77]. For clinicians, this heterogeneity implies that reported IAPA rates might be influenced by the diagnostic framework applied and local diagnostic practices. Consequently, incidence and outcome estimates should be interpreted in light of the specific criteria used, and cross-study comparisons require caution.

The structured design of this review, guided by PRISMA principles, ensured transparent and reproducible study selection and facilitated identification of major sources of heterogeneity. The use of robust descriptive statistics, heterogeneity testing, and diversity metrics allowed cautious interpretation of epidemiological estimates and supported the decision to avoid formal meta-analysis due to pronounced between-study variability.

To further describe the contribution of diagnostic frameworks to epidemiological variability, we performed stratified boxplot analyses of study-level incidence and case-fatality rates according to applied diagnostic definitions (Figs. 4 and 5). Visual inspection revealed numerical differences in central tendency across criteria, with higher median incidence estimates observed in studies applying EORTC/MSG- and Verweij-based definitions [2, 22, 72], whereas Blot-based definitions tended to yield lower incidence estimates [73]. For case-fatality rates, ICU-adapted definitions, particularly the Schauwvlieghe-based approach, showed numerically higher median mortality. However, overlap between criteria in studies applying multiple diagnostic definitions may reduce differences between criteria. In the multivariable linear regression analyses adjusting for diagnostic framework, none of the evaluated criteria demonstrated a statistically significant independent association with either IAPA incidence or case-fatality rate. For incidence, EORTC/MSG definitions showed the strongest positive trend (B = 9.126, *p* = 0.064), whereas for mortality, the largest effect estimate was observed for the Schauwvlieghe-based definition (B = 21.492, *p* = 0.058). Although these findings suggest potential directional effects, they did not reach conventional thresholds for statistical significance. The regression analyses were performed for exploratory purposes and should be interpreted cautiously given the heterogeneity across studies.

Collectively, these analyses indicate that while diagnostic definitions may influence case ascertainment patterns, they do not independently explain the pronounced inter-study heterogeneity. Rather, epidemiological variability likely reflects complex interactions between diagnostic strategy, screening intensity, patient selection, timing of disease recognition, and local ICU practice. These findings highlight the importance of harmonized, ICU-specific consensus definitions to enhance comparability and improve the interpretability of future epidemiological investigations.

To address this heterogeneity, we deliberately used robust descriptive statistical approaches, including trimmed medians and bootstrapped confidence intervals, rather than conventional pooled meta-analytic estimates. In combination with formal heterogeneity quantification using I² statistics and methodological diversity assessment, this approach allowed a more cautious interpretation of epidemiological estimates and reduced the influence of outlier studies. These methodological choices strengthen the validity of the conclusions and support our decision to avoid formal meta-analysis given the pronounced between-study variability. Additionally, the wide variation among incidence estimates observed across studies in the funnel plot, together with the results of Egger’s regression analysis, also suggests substantial inter-study heterogeneity. The influence of large-population studies with a highly different study approach on symmetry assessments indicates that methodological differences in included studies may contribute more to funnel plot asymmetry than true publication bias itself.

Despite growing awareness, IAPA remains a high-mortality complication of influenza. However, optimal preventive strategies remain uncertain, and available evidence is limited. IAPA is associated with mortality rates nearly twice as high as those observed in influenza patients without fungal superinfection [4, 5, 14]. Diagnosis remains challenging, as histological confirmation is rarely feasible in ICU patients and bronchoalveolar lavage for mycological testing is not always possible or feasible at admission [2, 75]. Delayed recognition may contribute to later initiation of antifungal therapy, which has been associated with poorer outcomes in invasive fungal infections.

The absence of temporal trends with respect to both publication year and median inclusion year, the latter serving as a proxy for the overall study period, further suggest that substantial improvements in the diagnosis and management of IAPA have not yet translated into measurable changes in epidemiologic parameters. These findings underline the need for continued research efforts and the development of improved diagnostic algorithms, biomarkers, therapeutic strategies, and risk stratification tools.

Two larger studies have evaluated antifungal prophylaxis or early empirical mold-active therapy in this setting [8, 9]. While both reported reductions in IAPA incidence, neither demonstrated a significant survival benefit. Taken together, these findings indicate that early antifungal strategies may reduce disease incidence but have not consistently demonstrated survival benefit, reinforcing the uncertainty surrounding optimal prophylactic approaches.

Based on currently available data, routine universal antifungal prophylaxis in influenza-associated critical illness cannot be recommended. Importantly, this conclusion primarily applies to indiscriminate, early universal prophylaxis administered to unselected critically ill influenza populations [8, 9]. However, this conclusion is constrained by the limited number of prospective studies and the heterogeneity of existing evidence.

One important consideration is the timing of disease onset. Many reported IAPA cases occur early, frequently at or shortly after ICU admission, which may limit the preventive effect of antifungal agents initiated at the time of ICU admission. Indeed, prophylaxis with antifungals, which often need days to reach steady-state drug concentrations, cannot avert infections that are already evolving at the time of initiation. In addition, breakthrough infections have been described in neutropenic patients despite therapeutic azole levels, suggesting that pharmacological prophylaxis may not be uniformly effective across heterogeneous influenza populations [62]. Indeed, the predominance of early-onset IAPA and the absence of a survival benefit in these existing studies challenge the biological plausibility of universal antifungal prophylaxis and highlight the risk of exposing large patient populations to treatment without clear clinical gain. Nevertheless, the precise impact of timing, drug pharmacokinetics, and host susceptibility on prophylactic efficacy remains insufficiently defined.

Another challenge is the marked heterogeneity in IAPA risk. Incidence, timing, and outcomes of IAPA vary widely between patient populations, indicating that susceptibility is driven by distinct host and disease factors rather than by influenza infection alone. Applying indiscriminate prophylaxis to populations with such diverse risk profiles dilutes potential benefit, increases drug exposure without clear justification, and may contribute to unnecessary toxicity, drug interactions, and antifungal resistance pressure. A recent systematic review and meta-analysis synthesized available data on the incidence and outcomes of influenza-associated pulmonary aspergillosis and reported pooled estimates suggesting that approximately one in five critically ill influenza patients develop IAPA, with a high associated fatality [14]. While these findings underscore the clinical relevance of IAPA, our review adds important contextual information by demonstrating the substantial variability in diagnostic definitions, study settings, and patient populations across studies, reflected by pronounced statistical heterogeneity and methodological diversity. Although meta-analytic techniques can provide summary estimates despite such heterogeneity, pooling across fundamentally diverse studies may result in overgeneralization. By deliberately applying robust descriptive estimators and systematically exploring heterogeneity, our analysis complements existing meta-analyses by identifying key sources of variability that need to be addressed before generalizable concepts or uniform preventive strategies can be derived.

Given the absence of survival benefit across studies and substantial methodological heterogeneity, universal antifungal prophylaxis cannot currently be recommended as standard practice. Future studies should focus on defining high-risk phenotypes, identifying patients with delayed susceptibility windows, and establishing validated triggers for targeted prophylaxis. A risk-adapted approach may represent a reasonable hypothesis, but it requires validation before clinical implementation. Given the limitations of universal antifungal prophylaxis, the combination of antifungal prophylaxis with immunomodulatory treatment targeted for selected high-risk patients may be a plausible solution [4, 11, 12]. However, these concepts remain investigational. Emerging data indicates that host genetic factors may contribute to differential IAPA susceptibility [13, 78]. For example, the *LGALS3* gene, encoding galectin-3, has been implicated in antifungal immune response, with Galectin-3 enhancing neutrophil recruitment and activity during *Aspergillus fumigatus* infection [79] and contributing to phagosome and lysosome repair [80]. In a preliminary cohort of 114 critically ill influenza patients, the AA genotype at rs4644 was associated with a significantly higher 30-day incidence of IAPA compared with AC/CC genotypes [81]. While hypothesis-generating, these findings require independent validation and mechanistic confirmation before clinical application. Building on the evidence summarized in this review, the Galectin-3 Targeted Antifungal Prophylaxis in ICU Influenza (GALActIC) project (EP PerMed Joint Transnational Call 2024 (PMTargets) Project ID: PIN1672624, https://galactic-project.eu/) represents a European multicentric translational step toward precision prevention of IAPA. It was initiated to further investigate the role of host genetic susceptibility in IAPA development. The project focuses on genetic variation in LGALS3, encoding galectin-3, which has been implicated in antifungal immune responses and has been associated in preliminary clinical data with differential IAPA risk.

GALActIC pursues four interlinked objectives: (i) validate the association between LGALS3 variants and IAPA susceptibility in independent international cohorts; (ii) characterize the functional consequences of galectin-3 variation on host immune responses to Aspergillus infection in the context of influenza; (iii) systems biology-based integration of transcriptomic, proteomic, metabolomic, and microbial datasets to map galectin-3–mediated signaling pathways and define IAPA risk profiles; and (iv) develop a clinical framework for evaluating whether LGALS3-guided risk stratification could inform targeted antifungal prophylaxis in future prospective interventional studies.

By integrating genetic validation with mechanistic and translational approaches, the project aims to determine whether host-directed risk stratification represents a feasible strategy for refining prevention concepts beyond universal prophylaxis. Any clinical implementation will require rigorous validation and confirmation in adequately powered randomized trials.

## Limitations

This review has several limitations. Substantial heterogeneity was observed across studies, reflecting differences in diagnostic definitions, patient selection, and ICU versus hospital-wide settings [2, 5, 22, 72, 73, 75]. The heterogeneity of applied criteria for IAPA, along with limited histopathological confirmation and variable bronchoalveolar lavage practices, may have led to misclassifications. In particular, the rate of BALF sampling has been shown to be strongly associated with incidence of COVID-associated pulmonary aspergillosis [75], and the same may be true for IAPA (for example 9% incidence with 44% BAL sampling [39] in comparison to 19% incidence with 54% BAL sampling [5], but the inconsistent reporting of BALF rates across IAPA studies did not allow for analyzing this potential association in more depth. Smaller studies disproportionately reported higher incidence, suggesting potential small-study or publication bias. Most studies were retrospective, and influenza confirmation methods varied, contributing to additional variability. In addition, this review was based on a structured literature search in a single bibliographic database, PubMed. While PubMed provides broad coverage of biomedical and infectious disease research, reliance on a single database may have resulted in incomplete retrieval of relevant studies, particularly those indexed exclusively in other databases, regional sources, or the grey literature. Another limitation is that study screening and selection were performed by a single reviewer. This deviates from standard systematic review methodology, which typically recommends independent screening by at least two reviewers to minimize selection bias and errors in study inclusion or exclusion. Although eligibility criteria were predefined and consistently applied, and study inclusion and exclusion were checked in detail by the shared first and senior authors as well as co-authors, the possibility of missed eligible studies or subjective selection decisions cannot be fully excluded. Importantly, due to the narrowly defined scope of this review, studies reporting data on IAPA in influenza populations were included irrespective of study design or setting, which limited the degree of discretionary judgment during the selection process. Furthermore, no formal study-level risk-of-bias or quality assessment was performed. Given the predominance of retrospective observational studies, heterogeneous study designs, and inconsistent diagnostic criteria, the application of a single standardized risk-of-bias tool was considered methodologically challenging and of limited interpretability. As a result, differences in internal validity, confounding, and outcome ascertainment across studies could not be systematically weighted or adjusted for and may have influenced the reported incidence and fatality estimates. Due to these circumstances, selection bias, detection bias or confounding cannot be excluded in the included studies. The absence of a formal quality assessment therefore limits the ability to contextualize individual study findings and underscores that the results should be interpreted as descriptive summaries of the available literature rather than as pooled effect estimates based on graded evidence.

Studies relying on ICD-based classification may be subject to misclassification bias [75]. Although, in clinical practice, established diagnostic approaches are typically used to distinguish colonization from infection, this differentiation is not transparent in administrative coding systems. Variability in clinical and coding practice across institutions may therefore contribute to potential misclassification of aspergillosis.

## Conclusion

Across 53 studies, IAPA represented a frequent and severe complication of severe influenza, with a trimmed median incidence of approximately 15% and a case-fatality rate of 50%. Universal antifungal prophylaxis has not shown significant survival benefits. Our findings argue against a one-size-fits-all approach and instead support the development of genetically informed, targeted prevention strategies. If validated, early LGALS3-guided risk stratification could enable timely, individualized prophylaxis, reduce unnecessary antifungal exposure, and ultimately improve survival in high-risk ICU patients. The GALActIC project exemplifies this shift toward precision prevention, addressing a critical clinical problem with a promising solution, aiming to integrate genetic testing into daily ICU practice to reduce mortality and improve patient outcomes.

## Supplementary Information

Supplementary Material 1.

Supplementary Material 2.

Supplementary Material 3.

## Data Availability

Not applicable.
