# Supplementary material for: Incidence and outcomes of influenza-associated pulmonary aspergillosis and the role of antifungal prophylaxis: a structured literature review
Source: Crit Care. 2026 Mar 26;30:231. doi: 10.1186/s13054-026-05975-1 (PMC13141606; doi:10.1186/s13054-026-05975-1)
Supplement: Supplementary file 1 — Supplementary Material 1. [file 13054_2026_5975_MOESM1_ESM.docx]

**Search strategy**

(("Influenza, Human"[Mesh]

OR "Influenzavirus A"[Mesh]

OR "Influenzavirus B"[Mesh]

OR influenza[All Fields]

OR flu[All Fields]

OR "influenza virus"[All Fields]

OR "influenza viruses"[All Fields]

OR "influenza A"[All Fields]

OR "influenza B"[All Fields]

OR "seasonal influenza"[All Fields]

OR "pandemic influenza"[All Fields]

OR H1N1[All Fields]

OR H3N2[All Fields]

OR H5N1[All Fields]

OR ILI[All Fields]

OR "influenza-like illness"[All Fields]

OR "avian influenza"[All Fields]

OR "swine flu"[All Fields]

OR "post-influenza"[All Fields]

OR "influenza infection"[All Fields]

OR "influenza infections"[All Fields])

AND

("Aspergillosis"[Mesh]

OR "Aspergillosis, Pulmonary"[Mesh]

OR "Aspergillus Infections"[Mesh]

OR aspergillosis[All Fields]

OR aspergilloses[All Fields]

OR "pulmonary aspergillosis"[All Fields]

OR "invasive aspergillosis"[All Fields]

OR "invasive pulmonary aspergillosis"[All Fields]

OR "aspergillus infection"[All Fields]

OR "aspergillus infections"[All Fields]

OR "tracheobronchial aspergillosis"[All Fields]

OR "aspergillosis-associated pulmonary pathology"[All Fields]

OR "aspergillosis-associated pulmonary process"[All Fields]

OR "Aspergillus fumigatus"[All Fields]

OR "Aspergillus species"[All Fields]

OR "Aspergillus spp."[All Fields]

OR IPA[Title/Abstract]))

OR

("IAPA"[All Fields]

OR "IAPA syndrome"[All Fields]

OR "influenza-associated pulmonary aspergillosis"[All Fields] OR "influenza associated pulmonary aspergillosis"[All Fields] OR "pulmonary aspergillosis associated with influenza"[All Fields] OR "post-influenza aspergillosis"[All Fields])

AND

("2000/01/01"[Date - Publication] : "2025/06/30"[Date - Publication])

NOT (

"Haemophilus influenzae"[All Fields]

OR "H. influenzae"[All Fields]

OR "h influenzae"[All Fields]

OR "Haemophilus-influenzae"[All Fields]

OR "Haemophilus influenza"[All Fields]

OR "haemophilus influenza infection"[All Fields]

OR "haemophilus influenza infections"[All Fields]

OR "iApa-I"[All Fields]

OR "iapai"[All Fields]

OR "IA-PA"[All Fields])
